# Spinal injury with spinal ankylosing disorders as a primary cause of death: report of two cases

**DOI:** 10.1186/s12245-023-00488-y

**Published:** 2023-02-16

**Authors:** Takahito Miyake, Hideshi Okada, Norihide Kanda, Yosuke Mizuno, Kodai Suzuki, Tomoaki Doi, Takahiro Yoshida, Shozo Yoshida, Shinji Ogura

**Affiliations:** 1 Advanced Critical Care Center, Gifu University Hospital, 1-1 Yanagido, Gifu, 501-1194 Japan; 2 Department of Emergency and Disaster Medicine, Gifu University Graduate School of Medicine, 1-1 Yanagido, Gifu, 501-1194 Japan; 3 Department of Abuse Prevention Emergency Medicine, Gifu University Graduate School of Medicine, Gifu, 501-1194 Japan

**Keywords:** Spine ankylosing disorders, Hemothorax, Hemorrhagic shock

## Abstract

**Background:**

Spinal ankylosing disorders (SADs) refer to a group of conditions resulting in spontaneous or postsurgical ossification and fusion of the spinal segments. The spine becomes increasingly susceptible to injury over time such that even low-energy trauma can cause a spinal injury. We report two cases of SADs, associated with massive thoracic hemorrhage.

**Case presentation:**

The first patient was an 85-year-old male, who suffered from a vehicular crash. He was diagnosed with a fracture of the first lumbar vertebra, accompanied by SADs. Intubation was required, and thoracic drainage tubes were inserted. The patient underwent a massive transfusion and thoracotomy with packing. Despite prompt treatment, the hemorrhage from the vertebral fracture was uncontrolled, and the patient died 180 min after the injury.

The second case features an 88-year-old male who fell from a height. He was diagnosed with flail chest, hemothorax, pneumothorax, and a fracture of the eighth thoracic vertebra with SADs. After intubation, four thoracic drainage tubes were placed, and a massive transfusion was conducted. He died after 3 days due to hypoxemia secondary to persistent bleeding of the vertebral fracture for 24 h.

**Conclusions:**

The patients died of persistent thoracic hemorrhage, and the sources of bleeding were the fracture site of the spine fractures. Controlling spinal hemorrhage is difficult due to the absence of a bleeding artery, which is managed via trans-arterial embolization. This report emphasized that fracture of SADs could be a fatal disease that requires prompt intervention.

## Background

Spine ankylosing disorders (SADs) consist of three major entities, namely ankylosing spondylitis (AS), disseminated idiopathic skeletal hyperostosis, and “end-stage advanced spondylosis multiforme” [1]. These entities have also been described as “rigid spine” diseases because they induce spontaneous or postsurgical ossification and fusion of the spinal segments [2]. Prior multi-level spontaneous inter-vertebral fusion in these cases results in vulnerability for major fracture-dislocation and distraction following relatively minor vertebral column impact [3]. In addition, from the epidemiology data, some SADs express osteoporotic bone and have a high incidence in elderly men [2]. Due to the lack of understanding regarding the clinical course of SADs, its delayed diagnosis in the primary, emergency, and radiologic fields has been commonly reported [2]. We report two cases of SADs, associated with massive thoracic hemorrhage.

### Case presentation

#### Case 1

**Fig. 1:**
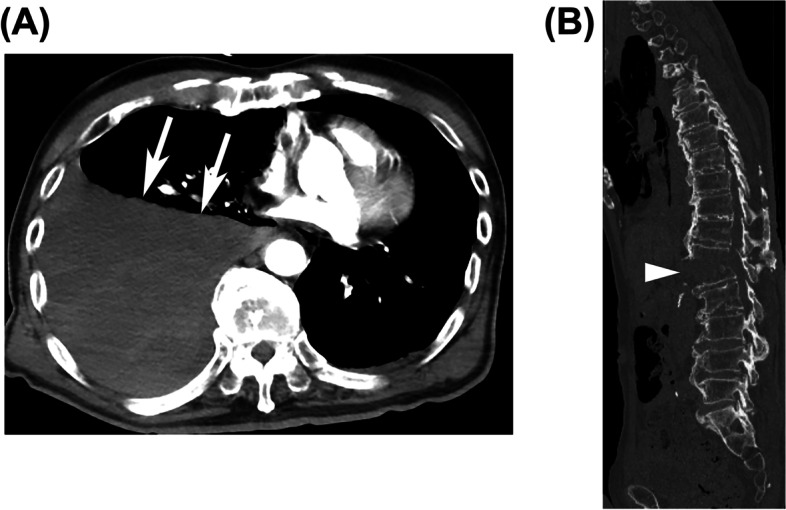
Computed tomography (CT) images of case 1 in the emergency department. **A** Enhanced axial CT revealed massive right hemothorax (arrow). Blood clots that appear to be associated with major blood vessels such as the arteries or vessels, including the vena cava. **B** Sagittal CT revealed a fracture of the first lumbar vertebra (triangle). The fracture site was widely opened. Notably, there was excessive bone bleeding potentiated by distraction and dislocation of the vertebral body, as indicated in **B**. Fractures of spine ankylosing disorders are considered “long bone fractures”

An 85-year-old man, who suffered from a vehicular crash, was transported to our hospital. On arrival, he had stable vital signs with a respiratory rate of 19 breaths per minute, oxygen saturation of 100% (10 L/min oxygen), blood pressure of 106/85 mmHg, pulse rate of 80 beats per minute, Glasgow Coma Scale (GCS) of E2V2M4, and body temperature of 36.5°C. His neurological symptoms of extremities could not be assessed. He was diagnosed with a fracture of the first lumbar vertebra, SADs, and hemothorax of the right lung based on the computed tomography (CT) scan results (Fig. 1A, B). Notably, he also had excessive bone bleeding potentiated by distraction and dislocation of the vertebral body, as indicated in Fig. 1B. He had an injury severity score (ISS) of 16. Laboratory testing (normal ranges in parentheses) at the emergency department (ED) revealed a white blood cell count of 8.06 × 10^3^/μL (3.3–8.6 × 10^3^/μL), serum hemoglobin of 12.3 mg/dL (13.7–16.8 mg/dL), platelet count of 168 × 10^3^/μL (158–348 × 10^3^/μL), serum creatinine kinase of 127 U/L (59–248 U/L), partial thromboplastin time of 32.9 s (25–38 s), international normalized ratio of 1.00, prothrombin time of 13.8 s (9.8–12.1 s), serum fibrinogen of 122 mg/dL (200–400 mg/dL), serum D-dimer of 37.8 μg/mL (<1.0 μg/mL), and serum fibrinogen degradation products (FDPs) of 61.1 μg/mL (<5.0 μg/mL). Venous blood gas analysis revealed acidosis and increased serum lactate (FiO_2_, 1.0; pH, 7.270; pCO_2_, 60.6 Torr; pO_2_ 27.2 Torr; HCO_3_, 26.9 mmol/L; base excess -0.6; and lactate, 30 mg/dL).

After undergoing an enhanced CT scan, the patient was intubated, and a thoracic tube was inserted due to a massive thoracic hemorrhage. Over 1000 mL was drained, and a massive transfusion was conducted. A right thoracotomy was performed 20 min after thoracic tube drainage because the patient remained hemodynamically unstable. The enhanced CT scan revealed that there was no arterial disruption at the para-spinal area, and the origin of the bleeding was mainly the fracture site, and thoracic packing was performed within 15 min. Despite these, the bleeding was uncontrolled, and the patient went into cardiopulmonary arrest. He died 3 h after ED admission.

#### Case 2

**Fig. 2:**
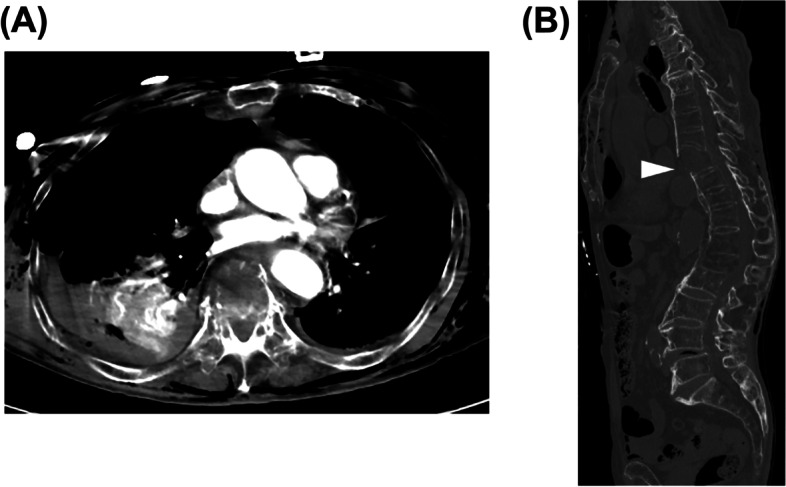
Computed tomography (CT) images of case 2 in the emergency department. **A** Enhanced axial CT showed massive right hemothorax, even after thoracic tube drainage. **B** Sagittal CT showed a fracture of the eighth thoracic vertebra (triangle). The fracture site was widely opened

An 88-year-old male was injured due to a fall from a height. Upon arriving at the hospital, he was intubated, and the drainage of the pneumohemothorax was conducted by the air ambulance team. He had a respiratory rate of 25 breaths per minute, oxygen saturation of 100% (FiO_2_ 1.0), systolic blood pressure of 66/46 mmHg, pulse rate of 91 beats per minute, GCS of E4V4M6 without obvious neurological deficits of lower extremities, and body temperature of 37.1°C. He was diagnosed with a vertebral fracture at the level of the first and eighth thoracic vertebrae with SADs. A right hemothorax and flail chest were also seen on the CT scan (Fig. 2). There were no signs of arterial bleeding on the enhanced CT scan. He had an ISS score of 25. Laboratory testing (normal ranges in parentheses) at the ED showed a white blood cell count of 20.13 × 10^3^/μL (3.3–8.6 × 10^3^/μL), serum hemoglobin of 9.5 mg/dL (13.7–16.8 mg/dL), platelet count of 181 × 10^3^/μL (158–348 × 10^3^/μL), serum creatinine kinase of 216 U/L (59–248 U/L), partial thromboplastin time of 59.4 s (25–38 s), international normalized ratio of 1.35, prothrombin time of 16.1 s (9.8–12.1 s), serum fibrinogen of 128 mg/dL (200–400 mg/dL), serum D-dimer of 372.9 μg/mL (<1.0 μg/mL), and serum FDPs of 1096.7.1 μg/mL (<5.0 μg/mL). Arterial blood gas analysis revealed mixed acidosis with an elevated serum lactate level (FiO_2_, 0.50; pH, 7.308; pCO_2_, 41.7 Torr; pO_2_ 147.0 Torr; HCO_3_, 20.3 mmol/L; base excess -5.1; and lactate, 45 mg/dL).

An enhanced CT scan indicated that there was no arterial disruption in the para-spinal area. Four thoracic tubes were placed to drain the hemorrhage, and a massive transfusion was initiated. Although it was difficult to estimate the exact position of the bleeding point, hemorrhage was mainly from the fracture site, and some from the lung contusion.

**Fig. 3:**
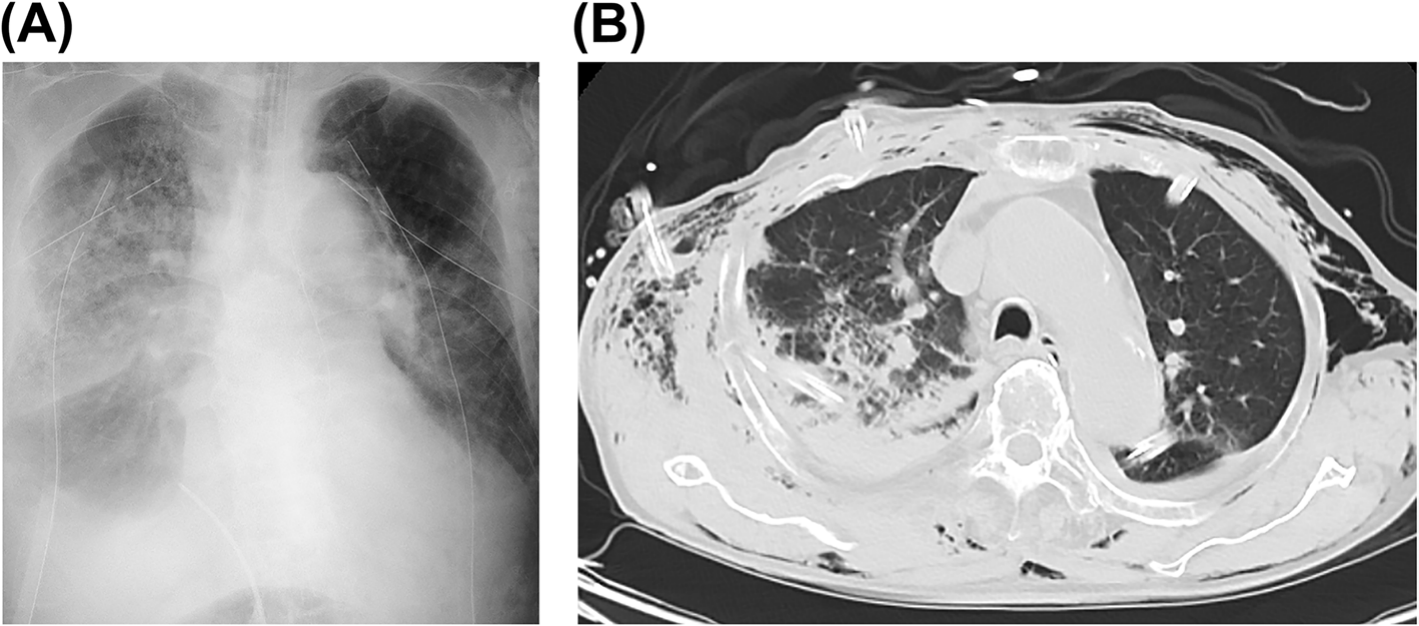
Chest X-ray and computed tomography (CT) images of case 2 on day 2. **A** Chest X-ray showed a right-sided consolidation with thoracic fluid. **B** Axial chest CT showed a right-sided dorsal consolidation with thoracic fluid. Active thoracic bleeding was stopped, per the findings of drainage tubes and CT images

After 24 h of transfusion, active bleeding was controlled, but the patient remained severely hypoxic. The chest X-ray and CT scan revealed the acute respiratory distress syndrome directly and indirectly from severe thoracic trauma and systematic inflammation (Fig. 3A, B). The patient died due to pulmonary distress 3 days after his ED admission.

## Discussion and conclusions

Both patients died of persistent thoracic hemorrhage, and the spinal fracture sites were the identified sources of bleeding. These cases demonstrated the difficulty of controlling thoracic hemorrhage from the fracture site. Fractures secondary to SADs are fatal due to two reasons.

### Biomechanical features of SADs

SADs are characterized by multilevel bony fusion and a long lever arm [3, 4]. Therefore, the spine becomes increasingly susceptible to injury over time such that low-energy trauma can cause spinal injury. The development of neurological deficits among patients with spinal injuries and SADs has been reported [2–5]. This indicates the instability of these types of fractures. According to previous studies, epidural hematomas result in delayed neurological deterioration [3, 5]. Shah et al. described the mechanical instability of a “rigid spine.” The biomechanics are altered due to the compromised spine-stabilizing components [2]. The altered biomechanics and long lever arm make the spine prone to a fracture even after low-energy trauma. There have been reports of patients with AS or severe arthrosis, who suffered from traumatic aortic rupture [6, 7]. The mechanism of injury involved significant adhesions between the aorta and anterior longitudinal ligament. Based on these, SAD fractures injure the small or large vessels surrounding the spine.

Based on the biomechanical features of SADs, ankylosing spinal fractures are considered “long bone fractures” (Fig. 1b). Based on a previous report, the estimated blood loss of a patient suffering from a femoral shaft fracture exceeded 1000 mL [8]. Since the spine has a higher degree of vascularity than the femur, massive hemorrhage is expected in cases of thoracic trauma.

### Limitation of hemostasis technique

The blood supply of the thoracic spine is mainly from intercostal arteries and that of the lumbar spine is from the lumbar and iliolumbar arteries [9]. When a thoracolumbar injury occurs, disruption of the thoracic or abdominal aorta, intercostal artery, and lumbar arteries could happen [10].

Identifying and controlling aortic or inter-costal artery bleeding sources during surgery or by trans-arterial embolization (TAE) and selecting the surgical approach for thoracotomy are difficult. If the bleeding source is arteries around the vertebrae (e.g., lumbar artery), hemostasis is achieved via TAE [7, 11]. In the orthopedic oncology setting, there have been reports of arterial embolization pre-operatively in spinal metastasis cases, which can reduce bleeding from the surgical site [12]. In the reported cases, arterial embolization might indirectly reduce the blood flow to the fracture site, which could be advantageous. However, in cases where the source of bleeding is the vein or a fracture site, achieving sufficient hemostasis is difficult. In that case, gauze packing or reduction of the fracture could be a choice. Unlike the femur, the spine is in contact with huge spaces, such as the thorax; thus, packing becomes less effective. Introducing a pillow under the thoracic spine was reportedly effective in closing the fracture site, particularly in patients with AS who have abnormal kyphosis [1]. We can also consider surgical fixation as an emergency treatment. Posterior fixation with the patient in the prone position could be first indicated for SAD; however, position change with unstable hemodynamics could be highly risky. Although the use of the bone cement to pack the vertebral fracture site for hemostasis and implant stabilization of the fracture with the anterior approach might be an option for stabilization, evidence of the hemostasis effect of bone cement is limited, and this option should be considered carefully [13].

In case 2, the hemorrhage was adequately controlled by transfusion therapy. However, severe respiratory problems, that were directly and indirectly associated with the massive transfusion, contusion, or hemothorax, were fatal. Both patients in this report shared the same cause of death.

SAD is commonly diagnosed late due to the lack of awareness of the disease and its impact on the patient’s condition [1]. Some cases with unclear causes of death were possibly due to hemorrhage secondary to fractured SADs. Therefore, early detection of injuries secondary to SADs is crucial to establish the appropriate strategy and to achieve adequate hemostasis.

Controlling hemorrhage secondary to SADs is difficult. TAE is not an appropriate intervention in these cases due to the lack of a bleeding artery. Hemorrhage from the fracture site is a fatal complication of SAD. Establishing immediate surgical hemostasis is critical in the management of hemorrhage secondary to injured patients with SADs.

## Data Availability

The datasets obtained and analyzed in the current study may be requested from the corresponding author.

## Abbreviations

AS: Ankylosis spondylitis
FDPs: Fibrinogen degradation products
ISS: Intensity severity score
CT: Computed tomography
ED: Emergency department
SADs: Spine ankylosing disorders
TAE: Trans-arterial embolization
